# The Role of Exosomes in Bone Remodeling: Implications for Bone Physiology and Disease

**DOI:** 10.1155/2019/9417914

**Published:** 2019-08-14

**Authors:** Christos Masaoutis, Stamatios Theocharis

**Affiliations:** First Department of Pathology, Medical School, National and Kapodistrian University of Athens, 11527 Athens, Greece

## Abstract

Bone remodeling represents a physiological phenomenon of continuous bone tissue renewal that requires fine orchestration of multiple cell types, which is critical for the understanding of bone disease but not yet clarified in precise detail. Exosomes, which are cell-secreted nanovesicles drawing increasing attention for their broad biosignaling functions, can shed new light on how multiple heterogeneous cells communicate for the purpose of bone remodeling. In the healthy bone, exosomes transmit signals favoring both bone synthesis and resorption, regulating the differentiation, recruitment, and activity of most cell types involved in bone remodeling and even assuming an active role in extracellular matrix mineralization. Additionally, in the ailing bone, they actively participate in pathogenic processes constituting also potential therapeutic agents and drug vectors. The present review summarizes the current knowledge on bone exosomes and bone remodeling in health and disease.

## 1. Introduction

Although grossly rigid and motionless, the bone is active, subject to an incessant, lifelong process of remodeling, i.e., renewal of aging, microdamaged tissue. This serves multiple purposes: bone maintenance or repair and adaptation to changing mechanical loads, as well as homeostasis of blood calcium and phosphorus levels. The bone remodeling seems to be triggered by osteocyte apoptosis, takes place simultaneously in multiple microscopic foci throughout the skeleton termed “bone remodeling compartments,” and requires the formation cell groups termed “bone multicellular units,” which are composed of chiefly three cell types: osteoclasts, osteoblasts, and osteocytes [1]. The osteoclasts originate from locally recruited monocytes following stimulation by osteoblast-derived receptor activator of NF-KB ligand (RANKL) and decompose old bone tissue [2]; the osteoblasts arise from mesenchymal stem cells and synthesize new mineralized extracellular matrix (ECM) [3]; the osteocytes are former osteoblasts entrapped inside the bone and possess mechanosensing properties [4]. The bone remodeling is governed by parathyroid hormone, vitamin D, and calcitonin and is largely impacted by growth hormone, estrogens, glucocorticoids, and thyroid hormones [5]. In the bone remodeling compartment microenvironment, the cell coordination implies complex mechanisms of intercellular communication not thoroughly elucidated yet.

Exosomes are cell-secreted, membrane-bound particles measuring 40-120 nm in diameter, which belong to “extracellular vesicles” along with microvesicles and apoptotic bodies [6]. Although they slightly overlap in size with the rest of the extracellular vesicles, their biogenesis is distinct and related to the endosomal pathway: an inward blebbing of the endosomal membrane produces intraluminal vesicles, which are then actively exocytosed as exosomes [7]. They carry a variety of biomolecules (proteins, nucleic acids, and lipids), which are compiled and readily searchable in digital libraries [8, 9]. Although their contents vary greatly depending on their cell of origin, all exosomes are equipped with endosomal proteins such as annexins, tetraspanins, and flotillin [10]. They function as intercellular mediators and are physiologically involved in immunity, coagulation, spermatogenesis, and central nervous system processes [11], whereas in cancer they mediate protumoral modifications of the tumor microenvironment and of remote premetastatic sites termed “premetastatic niches” [12]. Exosome isolation methods include differential centrifugation, considered as the “gold standard” technique [13], size exclusion [14], immunoaffinity isolation [15], polymeric precipitation [16], and the use of microfluidic devices [17].

**Figure 1:**
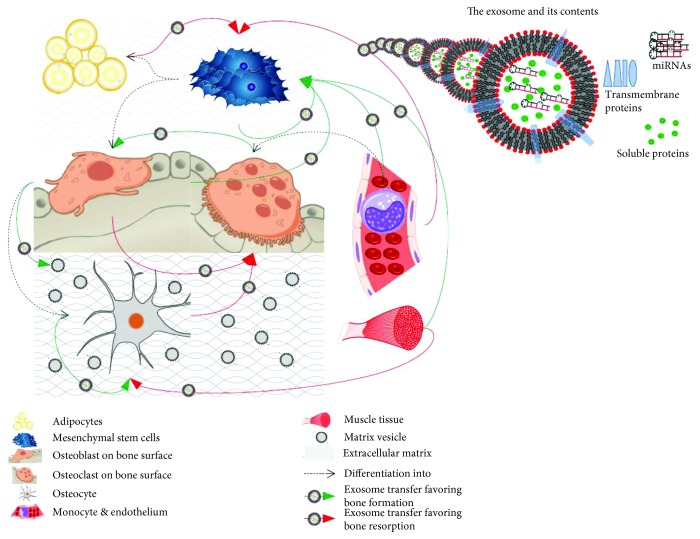
The role of exosomes in the intricate processes of physiological bone remodeling. In bone remodeling, exosomes are exchanged mainly among osteoblasts, osteocytes, and osteoclasts and their precursors and also secreted by adipocytes, myoblasts, and the endothelium (shown), as well as by dendritic cells and synovial fibroblasts (not shown). Some exosomes function as mineral nucleation sites (“mineralizing exosomes” or “matrix vesicles”). Exosomes carry a variety of biomolecules such as proteins and miRNAs (upper right corner), which favor either bone synthesis (green arrows) or bone resorption (red arrows) depending on the type of secreting and receiving cell.

Unlike the most extensive part of the literature on exosomes, which deals predominantly with cancer, we intend to feature their function in physiology and nonneoplastic pathophysiology of the bone. Recent reviews have elucidated their role in primary bone cancers [18] or in bone metastases [19, 20], which hence falls beyond the scope of the present paper. However, we exceptionally and briefly address the role of exosomes in multiple myeloma-related osteolysis, as the latter appears to be the result of a tumor-induced pathophysiological deregulation rather than tumoral invasion. In the first part of this paper, we aim to outline the role of exosomes in the intricate process of physiological bone remodeling, also illustrated in Figure 1 in a simplified manner. In the second part, we explore the potential usefulness of the exosomal model in the clinical setting with regard to therapy and/or understanding the pathophysiology of specific bone diseases.

## 2. Materials and Methods

We searched the computerized MEDLINE® database of the U.S. National Library of Medicine with the complex term *bone AND (exosome OR “extracellular vesicle”) AND (remodeling OR osteogenesis OR osteogenic OR “bone formation” OR osteoclast OR osteoblast OR fracture)*, which produced 148 results. When no reference to the skeletal system was made in the abstract, the article was excluded. The most common reason was the item *bone* appearing only as part of the term *bone marrow*. Articles referring to the teeth or—as explained above—cancer were also excluded. If the generic term “*extracellular vesicles*” instead of “*exosomes*” was used, the study was included only if isolation methods specific to exosomes had been applied. The paragraph dedicated to exosomes in the introduction was based on results from the relevant literature searches we conducted recently [18, 21]. Ultimately, the literature cited includes 119 peer-reviewed original articles and reviews published from 2001 up to 2019 in English. We used simple narrative analysis to summarize the data from the studies selected for review.

## 3. Exosomes in Bone Remodeling

The orchestration of bone remodeling is an archetypal complex physiological process highly dependent on finely tuned intercellular communication, which is only incompletely explained on the basis of the cytokine and the hormone model. Since their emergence as intercellular messengers, exosomes have come to disclose further details of bone physiology. Skeletal health is, certainly, also contingent upon successful angiogenesis and myogenesis, which are physiological processes also involving exosomal signaling. In the context of skeletal physiology, an abundance of proangiogenic (VEGF, transforming growth factor- (TGF-) *β*1, interleukin- (IL-) 8, hepatocyte growth factor (HGF), human T-cell factor 4 (TCF4), and multiple miRNAs) and promyogenic molecules (VEGF, IL-6, miR-494, and miR-181) has been found in exosomes, mostly shed by MSCs [22]. The following paragraphs are confined to the role of exosomes in bone remodeling, presented by the type of secreting cell.

### 3.1. Exosomes from Mesenchymal Stem Cells

As bone marrow MSCs differentiate into osteoblasts, their exosomal cargo is modified accordingly [23, 24]: some miRNAs are increased (let-7a, miR-199b, miR-218, miR-148a, miR-135b, miR-203, miR-219, miR-299-5p, and miR-302b) or decreased (miR-221, miR-155, miR-885-5p, miR-181a, and miR-320c), whereas some mRNAs (*ACIN1*, *DDX6*, *DGKA*, *DKK2*, *Lsm2*, *RPS2*, *Xsox17*, and the NF-*κ*B-related *ADAM17* and *NF-κB1*) are differentially expressed over time. These represent for the most part modulation of mRNA surveillance, Wnt signaling, and RNA transport pathways and also less prominent changes in numerous other intracellular pathways (i.e., biotin metabolism, RNA degradation, ubiquitin-mediated proteolysis, mTOR signaling, PI3K-Akt signaling, insulin signaling, aldosterone-regulated sodium reabsorption, MAPK signaling, tight junction, p53 signaling, focal adhesion, erbB signaling, B-cell receptor signaling, adipocytokine signaling, adherens junction, pantothenate and CoA biosynthesis, leukocyte transendothelial migration, valine, leucine, and isoleucine biosynthesis, gap junction, and VEGF signaling) [23].

Exosomes secreted by MSCs [24], either from the bone marrow [25, 26], the umbilical cord [27], or the adipose tissue [28] or from induced pluripotent stem cells [29, 30], can promote the osteoblastic differentiation of MSCs [25–27, 29, 30] or primary osteoblasts [28]. This is evidenced as upregulation of osteogenic genes [30] (*FGF2* [25], *BMP2* [25, 27], *GDF10* [25], *PHEX* [25], *ALPL* [25–27, 29], *TGF-β1* [25], *RUNX2* [25–29], osterix *(OSX)* [25, 27], osteocalcin *(OCN)* [25–27], *OPN* [25, 26, 28], *VEGFA* [25], *COL1* [25, 27–29], *BMP9* [25], *BMP6* [25], *GAPDH* [25], *B2M* [25], and *BSP* [28]) and increased osteoblast proliferation and migration [26, 28, 30]. The altered gene expression of MSCs following uptake of MSC-derived exosomes reflects the activation of pathways implicated in ECM-receptor interaction, cell adhesion, and PI3K-Akt signaling, the latter already associated with osteogenic differentiation [30]. Exosomes reinforce the osteoblastic differentiation of other MSCs also as part of a positive feedback loop. Those shed by MSCs already committed to an osteogenic phenotype (i.e., with early activation of *BMP2*, *OSX*, *SPP1*, *OSC*, *IBSP* (bone sialoprotein), and *ALP* (alkaline phosphatase)) can steer other MSCs towards the same phenotype [24, 31]. MSC-derived exosomes can also be directly uptaken by osteoblasts, promoting their proliferation and inducing the synthesis of GLUT3 and MAPK-pathway-related proteins [32].

Specific exosomal contents suggested to be responsible for their proosteogenic properties include miRNAs (miR-196a, miR-27a, and miR-206) [26] and, in the case of adipose tissue-derived MSCs, at least in the context of acute inflammatory bone injury, the Wnt-3a protein [28]. Thorough exosomal miRNA profiling and hierarchical clustering confirm their implication in pathways related with osteogenic differentiation, as well as in less specific pathways, e.g., related with Wnt signaling and endocytosis [24].

Conditioning with MSC-derived exosomes induces tissue changes, such as increased matrix mineralization [25, 27] and vascularization [25, 29], as well as bone regeneration in rat models of bone defects [26, 29, 30, 33]. Of note, MSC-derived exosomes bind directly to ECM proteins, such as type I collagen and fibronectin [25]. The promotion of matrix mineralization seems to be a feature of exosomes from cells in late—rather than early—stage osteoblastic differentiation [24].

Although all current evidence overwhelmingly supports that MSC-derived exosomes favor osteogenesis, an animal model study focused on alveolar bone deterioration showed that exosomes from bone marrow MSCs could enhance the differentiation of osteoclast precursor cells *in vitro* [34].

Lastly, exosomes seem to be also implicated in cartilage development. Bone marrow MSCs with an induced chondrogenic phenotype produce exosomes with an altered miRNA cargo (marked by an increase in miR-1246, miR-1290, miR-193a-5p, miR-320c, and miR-92a and a decrease in miR-377-3p and miR-6891-5p levels), which further favor chondrogenesis [35]. The most prominent chondrogenic effect is ascribed to exosomal miR-320c and is mediated by *SOX9* upregulation and metalloproteinase *MMP13* downregulation [35].

### 3.2. Exosomes from Osteoblasts

Ample data suggest that exosomes shed by osteoblasts occupy a role in bone physiology. They contain a wide range of proteins, the majority of which, unremarkably, participate in vesicle-related molecular processes, while others are more closely related to the functions of their parent cell (e.g., skeletal development, mesenchymal differentiation, calcium ion binding, and phosphatase activity) [36, 37]. For example, osteogenic proteins (bone morphogenetic proteins 1 to 7, alkaline phosphatase (ALP), and eukaryotic initiation factor 2 (EIF2)) and noncollagenous ECM proteins (bone sialoprotein, osteopontin, osteocalcin, and osteonectin) along with a variety of other proteins (annexins, peptidases, ion channels, 14-3-3 proteins, and Rab-related proteins) are packed inside exosomes derived from mineralizing osteoblasts [37]. Interestingly, a small subset of exosomal proteins is differentially expressed between mineralizing and nonmineralizing osteoblasts, as well as according to the stage of differentiation [36]. Ge et al.'s comprehensive proteomic analysis of osteoblast-derived exosomes concludes that their proteomic cargo implicates four cardinal osteogenesis-related pathways, namely, Rho GTPase binding, integrin, mTOR, and EIF2 signaling [38], and indicates key exosomal proteins, namely, ephrin-B1 (EFNB1), transforming growth factor beta receptor 3 (TGFBR3), low-density lipoprotein receptor-related protein 6 (LRP6), bone morphogenetic protein receptor type 1 (BMPR1), and SMURF1 [39]. These pathways and proteins are involved in both bone synthesis and bone resorption [39].

Exosome-mediated mechanisms favoring bone synthesis involve miRNAs [40] and the transforming growth factor beta receptor II interacting protein 1 (TRIP-1) [41]. In the first case, exosomes from mineralizing osteoblasts are capable of shifting the recipient MSC miRNA profile (particularly miR-3084-3p, miR-680, miR-677-3p, and miR-5100) towards osteoblastic differentiation via Wnt activation and Axin1 inhibition, presumably through exosomal miRNA delivery (particularly of miR-667-3p, miR-6769b-5p, miR-7044-5p, miR-7668-3p, and miR-874-3p) [40]. In the second case, osteoblasts transport TRIP-1 to the ECM via exosomes, a protein that binds to type 1 collagen and boosts matrix mineralization through calcium and phosphate deposition, collagen fibril arrangement, and Runx2 and alkaline phosphatase activity [41].

On the contrary, TRAP [42], RANKL [42, 43], and osteoprotegerin [42], factors with established proosteoclastic functions, are also secreted by osteoblasts in a vesicle-bound form with [43] or without [42] prior parathyroid hormone stimulation. The RANKL of extracellular vesicles interacts with the RANK of osteoclasts or osteoclast precursors and can induce the differentiation of the latter [43]. The RANK-RANKL binding occurs on the surface of the extracellular vesicle and the target cell rather than by fusion of the vesicle with the target cell plasma membrane [43].

### 3.3. Exosomes from Osteocytes

Although traditionally perceived as relatively inert and trapped in bone lacunae, osteocytes remain active and, what is more, employ exosomes in order to perform not only paracrine but also systemic functions. In fact, they possess cytoplasmic projections reaching the vascular-facing surface of the osteoblast layer [44], which could be how they release exosomes into the circulation [45]. These exosomes are hypothesized to contain high levels of specific miRNAs (miR-3473a, miR-3473b, miR-3473e, miR-5128, miR-6244, miR-6239, miR-5132-5p, miR-705, miR-208a-5p, miR-3104-5p, miR-1224-5p, and miR-5621-5p) and alter the overall circulating exosomal miRNA profile [45].

An exosome-related mechanism has been proposed to elucidate how mechanical stimuli activate bone synthesis, based on the observation that mechanically induced calcium oscillations bring about not only actomyosin contractions in the osteocyte's cytoskeleton but also increased exosome release [46]. Other data indicate that osteocyte-derived exosomes mediate bone resorption. On the one hand, osteocytes produce vesicles containing osteoclastogenesis-regulating factors TRAP, RANKL, and osteoprotegerin, as osteoblasts do [42] (see Section 3.2). On the other hand, osteocyte-derived exosomes produced after stimulation with myostatin, a myokine, inhibit osteoblastic differentiation (lowering Runx2 levels and downregulating the Wnt signaling pathway), probably through miR-218 [47].

### 3.4. Exosomes from Osteoclasts

Osteoclast-derived exosomes seem to have an overall proosteoblastic effect, witnessed as overexpression of *Runx2* in osteoblasts and increase in calcium salt deposition [48]. A subset thereof carries high concentrations of RANK [49, 50], speculated to act in a dual fashion: firstly, by binding to secreted RANKL before it reaches the osteoclast surface and thus sparing the osteoclasts from RANK activation [49], a phenomenon also occurring after the administration of antiosteoporotic drug clodronate [51] (see Section 4), and secondly, possibly by binding to RANKL-bearing cells so as to transfer other regulatory molecules [49]. Notable exosomal contents besides RANK are miRNAs (particularly miR-146a [50] and 214–3p [50, 52, 53]), semaphorin 4D [50], ephrin-A2 [50], and RANKL [50], which exhibit the following properties: miR-214-3p is taken up by osteoblasts, hinders osteogenesis [52] apparently through osterix and activating transcription factor 4 (ATF4) regulation [54], and can be inhibited by antagomir-214-3p [52]; semaphorin 4D is necessary for the interaction between osteoclast-derived exosomes and osteoblasts *in vitro* [52]; ephrin-A2, a member of the ephrin family of proteins, implicated in bone remodeling [55], binds to its receptor EphA2 on the surface of osteoblasts [53].

Exosomes shed by mature osteoclasts differ significantly from those shed by osteoclast precursors, in that only the former contain RANK [49] and inhibit osteoclast formation [50], whereas the latter stimulate osteoclast proliferation [50].

### 3.5. Exosomes from Monocytes and Dendritic Cells

Monocyte-derived exosomes exert proosteogenic effects on MSCs. They induce the expression of *Runx2* [56], *BMP2* [56], and matrix metalloproteinase genes *MMP3* and *MMP1* [57] but not osteocalcin [56] and boost the production of cytokines CXCL5, CXCL3, and interleukin-1 [57]. These changes are probably mediated through miRNA delivery [56].

Dendritic-cell-derived exosomes carry a variety of chemotactic agents and can recruit MSCs [58], a crucial step in tissue regeneration. However, evidence on whether they favor osteoblastic differentiation is conflicting: Silva et al. noted no evidence of exosome-induced MSC differentiation [58], whereas Wang et al. demonstrated *Runx2* overexpression and an increase in ALK activity following MSC treatment with exosomes harvested from dendritic cells [59].

### 3.6. Exosomes from Other Cell Types

Osteoblasts and mature fat lie in close proximity and share a common progenitor, the MSC, whose differentiation can be shifted either way under the influence of various factors [60], in part as a result of a fat-osteoblast crosstalk via exosomes. More specifically, adipocytes deliver to MSCs RNA transcripts (of *PPARγ*, *CEBPα*, and *CEBPδ*) and miRNAs (miR-138, miR30c, miR125a, miR-125b, and miR-31) that target the transcripts of osteogenic genes (*Runx2*, *Osterix*, *Smad2*, and *Smad4)* and cause downregulation of osteocalcin (OSC) and osteopontin, all indications of an antiosteogenic impact [61]. Preadipocytes may favor each other's osteogenic differentiation through the exchange of exosomes which downregulate miR-223 in the recipient cell [62]. Exosomes from sinus mucosa cells, which line the osseous cavities, also promote osteoblastic differentiation and bone regeneration *in vitro* and *in vivo* [63]. Normal synovial fibroblasts can effect changes in articular chondrocytes and human umbilical vein endothelial cells via exosomes. These changes were more pronounced when the synovial fibroblasts were treated with IL-1*β*, mimicking the pathology of osteoarthritis, and include, in the case of chondrocytes, upregulation of *MMP13* and *ADAMTS5* and downregulation of *COL2A1* and *ACAN* and in the case of endothelial cells increased migration and tube formation activity [64]. Conversely, the endothelium, when senescent, delivers miR-31 via exosomes to MSCs, where it inhibits the Wnt pathway-related protein Frizzled-3 and thereupon osteogenic differentiation [65]. Nonsenescent endothelial cells, however, secrete exosomes that inhibit osteoclastogenesis and attenuate bone resorption via miR-155 upregulation [66]. Exosomes produced by endothelial progenitor cells contain lncRNA-MALAT1, which binds to and inhibits miR-124, and therefore promote osteoclastic differentiation of bone marrow-derived macrophages [67]. Myoblasts can convey miR-27-3p and other miRNAs (e.g., miR-206) to preosteoblasts via exosomes. In the preosteoblasts, miR-27a-3p targets the *APC* gene and consequently activates the *β*-catenin pathway, a critical intracellular event for their differentiation into osteoblasts [68]. Indirect muscle-bone communication has also been described, i.e., in the form of myostatin-induced production of antiosteogenic exosomes from osteocytes (see Section 3.3) [47].

### 3.7. Exosomes and Matrix Vesicles

Matrix vesicles are small, osteoblast-secreted vesicular particles enveloped by a lipid bilayer membrane, critical for the formation of calcifying nodules in primary mineralization [69, 70]. They are not mere bone mineral nucleation sites, but biologically active bodies equipped with a variety of membrane transporters and enzymes [69], even responsive to vitamin D metabolites [71]. Shapiro et al. provide plausible evidence that matrix vesicles are actually osteoblast-secreted exosomes anchored to the extracellular matrix, arguing that they are largely homologous in terms of size, composition, and biosynthesis (although matrix vesicles have adhesive properties, while exosomes typically do not) [72]. In fact, they discriminate between two main pathways of matrix vesicle biosynthesis: the first is identical to exosome synthesis and produces what the authors term “mineralizing exosomes,” whereas the second involves mineral-nuclei-containing autophagosomes that are exocytosed in the form of what the authors term “mineralizing ectosomes” [72]. This concept integrates matrix vesicles into the exosome model of cell-cell and cell-matrix interactions.

## 4. Potential Clinical Implications

Exosome-based intercellular communication is pivotal to the efficient orchestration of bone tissue repair. Mice with impaired exosome formation (CD9 knockout) also exhibit defective fracture repair in terms of chondrocyte and woven bone formation, vascularization, and healing time, all largely attributable to the lack of MSC-produced exosomes [73].

Exosomes, engineered or not, can be used to enhance bioactive materials for therapeutic purposes [22, 74]. The combinations of exosomes with tricalcium phosphate (*β*-TCP) [30, 75] or poly(lactic-co-glycolic acid) (PLGA) [75] scaffolds, i.e., bone graft substitutes, allow for the slow release of exosomes into the regenerating bone tissue [30] and their subsequent uptake by bone marrow MSCs [30] with multiple favorable results. These include the proliferation, migration, and osteogenic differentiation of MSCs [30, 75], alteration in gene expression involving the PI3K/Akt signaling pathway [30], and *in vivo* acceleration of bone regeneration [75]. Exosome-encapsulated titanium oxide nanotubes combined with osteoinductive protein BMP2 have also demonstrated favorable osteogenic properties [76].

In addition, exosomes illuminate parts of the mechanism of action of some osteoprotective drugs. Clodronate, a bisphosphonate, besides mediating proosteogenic molecular alterations involving ALP activity and *Runx2* and *Dlx5* genes, has been shown to promote the production of RANK-containing exosomes from bone marrow MSCs. These exosomes can foster osteoblastic differentiation [51]. Of note, RANK-containing exosomes are also thought to bind to extracellular RANKL before it comes to activate adjacent osteoblasts [49]. Liraglutide, an antidiabetic glucagon-like peptide-1 (GLP-1) analogue with a positive off-target impact on diabetic patients' bone health, alters the miRNA profile of bone-marrow-MSC-secreted exosomes. These miRNAs, besides targeting insulin secretion and insulin-signaling as expected, also alter the Wnt signaling pathway, which is crucial for bone remodeling [77].

In the following paragraphs, disease-specific data are presented regarding the role of exosomes in the development of new therapies for bone regeneration and the elucidation of pathophysiological aspects of bone disease.

### 4.1. The Aging Bone

Bone health deterioration is a universal feature of old age with a multifactorial etiology, largely involving osteoporosis and osteoarthritis, which are discussed in more detail in Sections 4.2 and 4.3, respectively. Exosomes isolated from the aged bone marrow contain higher levels of the miR-183 cluster (miR-96, miR-182, and miR-183) [78] and of miR-31a-5p [79], which reduce MSC proliferation, osteogenic differentiation, and Hmox1 protein levels [78], induce MSC senescence [78], and promote osteoclastogenesis (i.e., RhoA activity) and bone resorption [79]. Aging-related effects were also reproduced by oxidative stress (i.e., H_2_O_2_ administration) [78]. Interestingly, miR-31a-5p can be silenced by antagomiR-31a-5p so as to lower osteoclastic activity and prevent bone loss, a potential therapeutic application [79]. The aged endothelium also exhibits altered exosomal miRNA composition, in the form of increased miR-31 levels. miR-31 can hinder osteogenic differentiation of MSCs by inhibiting Frizzled-3 protein and can be detected in the circulation of aged individuals [65].

### 4.2. Osteoporosis

Osteoporosis is a very common condition characterized by the loss of bone mass and alteration of bone architecture with consequent increased bone fragility and fracture risk [80]. Xie et al.'s comprehensive and quantitative proteomics analysis of circulating exosomes isolated from individuals with reduced bone mass supports the view that exosomes participate on the one hand in osteoclastogenesis and osteoclast activation and on the other hand in compensatory new bone synthesis [81]. Among the downregulated factors in circulating exosomes figure integrin-related proteins essential for mechanosensation and osteoblastic activation; in addition, the upregulated amyloid precursor protein (APP) and nucleolin (NCL) may facilitate osteoclast survival, although also the upregulated versican core protein (VCAN) and connective tissue growth factor (CTGF) may assist osteoblastic differentiation and adhesion [81]. Type 1 diabetes seems to compromise the proosteogenic properties of bone marrow MSC-derived exosomes, a finding possibly relevant to the pathogenesis of diabetes-related osteoporosis [82].

A multitude of miRNAs are deregulated in osteoporosis in a cell-free, vesicle-free circulating form [83]. However, these are not readily comparable with the exosomal miRNA cargo; some are not consistently up- or downregulated in animal models of osteoporotic fractures in their free circulating form (miR-140-3p, miR-214), while others seem to promote both osteoclastogenesis and osteoblast differentiation in their exosomal form (miR-148a, miR-218) [84]. Nonetheless, both free and exosomal miR-29b-3p seem to enhance mouse fracture healing [84]. miRNA-21, which has been found upregulated in circulating exosomes of osteoporotic individuals, has an antiosteogenic impact, in that it inhibits SMAD7 protein with a subsequent downregulation of *ALP*, *OCN*, and *RUNX2* [85]. Shen et al.'s study on transfer RNA-derived fragments (tRFs), a novel class of noncoding RNA [86], suggests that higher levels of circulating exosomal tRF-25, tRF-38, and tRF-18 predict a worse prognosis in osteoporosis with an estimated sensitivity and specificity in the order of 85%, based, however, on a relatively small number of 40 patients and equal number of controls [87].

Regarding potential therapeutic applications, studies on animal models demonstrated recently that endothelial progenitor cell-derived exosomes block osteoclast induction and inhibit osteoporosis via miR-155 [66] and promote bone regeneration by induction of angiogenesis in distraction osteogenesis via miR-125 [88]. In radiation-induced bone loss, specifically, exogenous MSC-derived exosomes can restore the osteogenic and adipogenic function of recipient MSCs, along with cellular parameters of radiation-related damage, via the Wnt/*β*-catenin signaling pathway [89].

### 4.3. Osteoarthritis

The exosome concept casts new light on the pathophysiology of osteoarthritis. Exosomes isolated from human synovial fluid carry miRNA profiles specific to osteoarthritis [64, 90] and, strangely enough, also to gender [90]. miR-504 seems to be particular to osteoarthritis irrespective of gender, though [90]. Exosomes secreted from synovial fibroblasts are taken in by articular chondrocytes [64, 90] and induce pathological cellular and molecular changes, such as downregulation of extracellular matrix synthesis genes *ACAN* (aggrecan) and *COL2A1* (collagen type II alpha 1) [64, 90], upregulation of *MMP13* and *ADAMTS-5* [64], and production of inflammatory cytokine interleukin-6 [90]. The above effects were exaggerated following the treatment of synovial fibroblast with interleukin-1*β* simulating the inflammatory milieu of osteoarthritis [64].

From a therapeutic point of view, exosomes produced by the bone marrow or embryonic MSCs can effect chondroprotective and anti-inflammatory changes in osteoarthritis according to data drawn from *in vitro* or animal models. In a way, MSC-derived exosomes seem to exert largely opposite effects compared to synovial fibroblast-derived ones. These effects include maintaining the synthesis-degradation equilibrium of chondrocyte ECM [91] or even accelerating cartilage repair [92] by increasing the production of cartilage ECM components (i.e., collagen type II [91–94], cartilage oligomeric matrix protein [92], aggrecan, a proteoglycan, [35, 93], and glycosaminoglycans [92, 94]) and decreasing the expression of MMP13 [93] and ADAMTS-5 in the presence of IL-1*β* [91, 93, 94]. Apart from ECM synthesis, a multifaceted mechanism is activated [92], which involves improved chondrocyte survival, proliferation, and migration related to the upregulation of *TGF-β1*, *Survivin*, *Bcl-2*, *FGF-2*, *PCNA* [92], and *SOX9* [35] and the activation of AKT and ERK pathways [92], as well as anti-inflammatory changes such as a reduction in IL-1*β* [92], TNF-*α* [92], and iNOS [93] and a shift to the “regenerative” M2 macrophage phenotype [92]. Protection from joint damage may also extend from the articular cartilage to the subchondral bone [94]. Exosomes derived from mature chondrocytes also help externally administered cartilage progenitor cells differentiate into stable cartilage through the TGF-*β*/SMAD pathway and/or *COL2A1* and *SOX9* upregulation, rather than produce foci of endochondral ossification [95]. However, this feature is more relevant to the repair of subcutaneous cartilage defects [95]. Many of the chondrogenic effects of MSC-derived exosomes are mediated by their rich miRNA cargo and, in particular, by miR-23b, miR-92a, miR-125b, miR-320, miR-145, miR-221, and miR-22, which therefore consist potential therapeutic factors [96]. The auspicious results of preclinical experiments summarized above suggest that MSC-derived exosomes could be a good cell-free alternative to MSC therapy, which is already being clinically tested, in terms of safety (being a nonpermanent, more easily suspended treatment, devoid of the risk of blood vessel occlusion or generation of inappropriate cell types), efficacy (being more amenable to process optimization), and cost effectiveness [96].

### 4.4. Autoimmune Diseases

Extracellular vesicles are known to participate in the pathogenesis of rheumatoid arthritis as carriers of autoantigens, proinflammatory proteins and miRNAs, and matrix degradation enzymes [97]. Exosomes, in particular and within the scope of pathological bone remodeling, have been studied in experimental models of rheumatoid and psoriatic arthritis. Synovial fibroblasts stimulated by TNF as in rheumatoid arthritis shed exosomes, whose miRNA content is implicated in both promotion and inhibition of bone formation, mainly by targeting inhibitors of BMP and Wnt pathways [98]. miR-221-3p in particular suppresses osteoblastic differentiation and maturation by lowering osteoblast Dkk2 expression [98]. Another miRNA also encountered inside synovial fluid exosomes, let-7b, plays a presumably vital role in rheumatoid arthritis because its GU-rich domain is essential for the Toll-like receptor 7 (TLR-7) to bind to an endogenous ligand on the surface of naive synovial fluid macrophages so as to transform them into inflammatory M1 macrophages [99]. The effect of circulating exosomes on osteoclast differentiation is inhibitory in the case of rheumatoid arthritis (as in healthy subjects) and stimulatory in the case of psoriatic arthritis [100] and is also appreciable in terms of *CALCR*, *CTSK*, and *RANK* gene expression. The latter stimulatory effect is not reproduced on monocytes from other individuals [100].

Exosomes derived from bone marrow dendritic cells possess anti-inflammatory and immunosuppressive properties, potentially exploitable for the treatment of inflammatory arthritis, based on a murine model of collagen-induced arthritis [101]. Periarticular administration of such exosomes mitigated delayed-type hypersensitivity responses within both injected and untreated contralateral joints, while systemic administration delayed the onset of the disease and tempered its severity [101].

Systemic lupus erythematosus (SLE), besides being a cause of inflammatory arthritis, is also characterized by impaired bone density and a higher fracture risk, a phenomenon with multifactorial, disease-specific, therapy-related, and comorbidity-related causes [102]. In a mouse SLE model with a homozygous *Fas^lpr^* mutation, exosomes were used to supply Fas-impaired cells with Fas protein so as to reduce intracellular miR-29b levels; this prevented Dnmt1-mediated hypermethylation of *Notch1* promoter and maintained the ability of MSCs to differentiate into osteoblasts. Thus, multiple parameters were improved, including trabecular bone volume, bone mineral density, and the bone volume/total volume ratio [103].

### 4.5. Avascular Bone Necrosis

Avascular bone necrosis, or osteonecrosis, is the result of diminished blood flow to the bone occurring in various diseases and is often a complication of medication, particularly of corticosteroids [104].

In an experimental model of hypoxia-induced bone damage, adipose tissue MSC-derived exosomes showed antiapoptotic effects attributable to the attenuation of reactive oxygen species (ROS) production, *Bcl-2* upregulation, *Bax* downregulation, and reduction of cytochrome c, cleaved caspase-9, and cleaved caspase-3 protein levels, as well as antiosteoclastogenic effects mediated by the modulation of the RANKL/OPG ratio [105].

In the case of corticosteroid-induced osteonecrosis, three exosome-based strategies have yielded positive results in animal models. The first strategy involves the engineering of exosomes that bear a mutated variant of hypoxia-inducible factor 1*α* (HIF-1*α*), which maintains function in normoxic conditions, from transfected bone marrow MSC. The exosome-bound mutant HIF-1*α* can increase trabecular reconstruction and microvascular density, indications of bone regeneration and angiogenesis [106]. The second strategy involves exosomes extracted from platelet-rich plasma, which can reinforce bone tissue resistance to glucocorticoid-induced apoptosis by inducing Bcl-2 expression through the Akt/Bad/Bcl-2 signal pathway, thus promoting bone tissue maintenance, regeneration, and cellular proliferation [107]. The third strategy involves bone marrow MSC-derived exosomes, which cause gene expression changes in the recipient MSCs relative to immune response, osteoblast differentiation, and the TGF-*β*/BMP signaling pathway, among which SOX9 upregulation might be the most vital [108].

### 4.6. Multiple Myeloma

Osteolysis, a clinical hallmark of multiple myeloma, results from changes in several intra- and intercellular pathways such as RANK/RANKL/osteoprotegerin, Notch, Wnt, RUNX2, EphrinB2/EphB4, and the TNF pathway, involving signaling molecules such as Dickkopf-1 (DKK1), sclerostin, periostin, osteopontin, growth factor independence-1 (GFI1), bone morphogenetic proteins, TGF-*β*, activin A, annexin II, adiponectin, Bruton's tyrosine kinase (BTK), stromal cell-derived factor-1*α* (SDF-1*α*), chemokines, and interleukins [109]. Amid this complex intercellular crosstalk, exosomes secreted from myeloma cells participate in both the repression of osteogenesis [110, 111] and the promotion of osteoclastogenesis [111, 112]. On the one hand, they deliver DKK1 so as to downregulate *RUNX2*, *OSX*, and *COL1A1* in osteoblasts [111] and lncRNA RUNX2-AS1, an antisense strand of RUNX2, to MSC so as to inhibit *RUNX2* translation [110]. It is worth noting that, in this context, an inhibitor of exosome secretion, GW4869, could effectively prevent bone loss *in vivo* [110] and even increase cortical bone volume and sensitize myeloma cells to bortezomib [111]. On the other hand, they can increase CXC-chemokine receptor 4 expression in preosteoclasts so as to promote osteoclast differentiation [112] and enhance osteoclast activity [111].

## 5. Conclusions

The cell-to-cell communication for the coordination of bone remodeling occurs in part through exosomal exchange. Steering the MSCs towards or away from osteoblastic differentiation is pivotal in this regard [25–27, 29, 30, 40, 47, 59, 61, 65, 68]. However, many more exosome-mediated effects take place at the same time, such as regulation of osteoclastic differentiation and/or activity [34, 42, 43, 79] and matrix vesicle deposition [72]. Exosomes are also messengers of pathogenic signals in bone disease [64, 65, 78, 79, 90, 98, 100, 101, 110, 112]. Interestingly, exosomes relevant to bone remodeling are secreted not only by the protagonists of bone physiology, i.e., the osteoblasts/osteocytes and osteoclasts and their precursors, but also by various other cell types, such as dendritic cells [58, 59], adipocytes [61], synovial fibroblasts [64], the endothelium [65], and myoblasts [68], another indication of the complexity of bone remodeling.

In impaired bone repair, e.g., in fractures complicated by delayed healing or nonunion, the use of autologous bone graft is the “gold standard” practice [113], although limited by occasional donor-site morbidity issues [114], and is followed by allograft implantation [113] with known biocompatibility limitations. Ancillary therapeutic strategies in bone repair include biophysical enhancement (in the form of electromagnetic field or low-intensity pulsed ultrasound stimulation), locally applied agents (osteogenic materials, osteoconductive materials, tissue repair factors, and osteoinductive and morphogenetic factors), and systemically administered agents (e.g., parathyroid hormone, anti-sclerostin antibodies, or anti-DKK1 antibodies) with notable, yet suboptimal results [115].

Given the above disadvantages in current practice, exosomes constitute a potential therapeutic alternative. From a pharmacological point of view, the advantages of exosomes include the ability to carry at once multiple, both hydrophilic and lipophilic molecules, clinical safety [116], as well as biochemical stability *in vivo* [10] and in storage [117]. These biochemical properties also render exosomes potential vectors for gene therapy; this approach, however, has not been reported, yet, in the field of osteochondral regeneration [118]. In bone disease, in particular, they could be used as osteoprotective, proosteogenic, or antiosteoclastogenic agents per se [91, 93, 94, 101, 105, 107], enhancers of bone scaffolds [30, 75], vectors of drugs [103, 106] or nanomaterials [76], or even substitutes of MSC therapy [89, 96]. What is more, considering the osseous tissue as part of the more complex musculoskeletal system, exosomes could theoretically facilitate a more comprehensive approach to treating bone disease, which would involve the musculature and the nervous system; in fact, exosomes have been engineered so as to promote regeneration of muscle via angiogenic, antifibrotic, antiapoptotic, and myogenic effects [22, 74], tendon [119] or peripheral nerves via miR-133b delivery [74].

Current knowledge on exosomes in bone remodeling is based on data from almost exclusively preclinical experiments; clinical trials involving exosomes as diagnostic or therapeutic agents are practically limited to cancer and none has focused on bone repair [116]. Despite the lack of sufficient patient-based evidence, we believe that the ample data gathered thus far are convincing enough so as to further investigate the role and potential clinical utility of exosomes in bone remodeling for the purpose of personalized medicine.
